# Information-Theoretic Medical Image Encryption via LLE-Verified Chaotic Keystreams and DNA Diffusion

**DOI:** 10.3390/e27111149

**Published:** 2025-11-12

**Authors:** Ibrahim Al-dayel, Muhammad Faisal Nadeem, Yasir Bashir, Ayesha Shabbir

**Affiliations:** 1 Department of Mathematics and Statistics, College of Science, Imam Mohammad Ibn Saud Islamic University (IMSIU), Riyadh 11566, Saudi Arabia; iaaldayel@imamu.edu.sa; 2 Department of Mathematics, COMSATS University Islamabad, Lahore Campus, Lahore 54000, Pakistan; 3 Department of Mathematics, COMSATS University Islamabad, Wah Campus, Wah Cantt 47040, Pakistan; yasirbashir@ciitwah.edu.pk; 4 Department of Physical Sciences, The University of Chenab, Gujrat 50700, Pakistan; ayesha@phs.uchenab.edu.pk

**Keywords:** Shannon’s entropy, chaotic keystreams, Lyapunov exponent, DNA coding, permutation–diffusion, NPCR, UACI, information security, medical images

## Abstract

We propose an information-theoretic encryption scheme consisting of a four-dimensional chaotic map driver in combination with a prediction model using an LSTM neural net to generate a keystream, which was limited only after passing a test based on the largest Lyapunov exponent (LLE). Our security analysis used a permutation phase to remove spatial redundancy, which was followed by an invertible DNA cross-diffusion procedure based on RGB channels. The removal of uncertainty and redundancy was measured using Shannon’s entropy (7.99–8.00 bits per channel), pixel intercorrelation, and differential analysis (NPCR ≈ 99.6%, UACI ≈ 33.3%). In key space analysis (order ≈ 2^384^), self-right veneering with complete encryption validity was demonstrated in perfect decryptability. We explain how chaos verification enhances the statistical goodness of keystreams and provide ablations that separate each element’s influence on entropy and decorrelation.

## 1. Introduction

High-fidelity medical images (CT, MRI, and ultrasound) are integral to diagnosis, therapy planning, and telemedicine. As such data increasingly traverse hospital networks, clouds, and edge devices, confidentiality, integrity, and availability must be ensured without degrading clinical throughput. Generic ciphers are not always ideal for images because of strong inter-pixel correlations and structured redundancies, including exploitable spatial symmetry that invites statistical and differential attacks. Image-tailored cryptosystems, therefore, emphasize permutation diffusion pipelines, often driven by chaotic dynamics and/or bio-inspired operations in DNA domains, to disrupt spatial regularities and equalize histograms while preserving exact invertibility for decryption.

Chaos is attractive because of its sensitivity to initial conditions and ergodic, aperiodic behavior, which can be harnessed to generate keystreams and control permutation/diffusion. Foundational work has introduced classical and modern chaotic attractors and systems that continue to inform cipher design, including Rössler and Chen attractors, and four-dimensional and hyperchaotic variants [1,2,3]. Recent medical image-oriented schemes demonstrate how carefully constructed chaotic maps, traversal strategies, and cross-channel diffusion can raise security while meeting clinical constraints. For example, Josephus traversing and dynamic cross diffusion yield strong confusion/diffusion with patient privacy emphasis [4], while Fisher–Yates-style scrambling combined with filter-based diffusion further improves permutation balance and resistance to bias [5]. Hyperchaotic Josephus-based traversal and multi-band, multi-wing five-dimensional maps enriched with linear algebraic mixing extend the key space and unpredictability [6,7]. Concurrently, improvements in four-dimensional chaotic systems via evolutionary operators sharpen randomness and key sensitivity. Application directions include blockchain, backed cloud pipelines [8], region of interest (ROI) centric protection using chaotic S boxes [9], and lightweight schemes tailored to the Internet of Medical Things (IoMT) [10], with additional IoT-focused variants coupling novel hyperchaotic maps and DNA cubes [11].

Deep learning (DL) is increasingly fused with chaotic and DNA mechanisms to either synthesize keystreams or encode image features for secure transforms. Representative contributions include DeepKeyGen, a learned stream cipher generator for medical images; CNN-guided DNA encoding for encryption; and end-to-end feature encoding/decoding, which serve as the cryptographic substrate. Machine learning-aided hyperchaotic schemes (e.g., RBF-based designs) and improved GAN-driven double encryption further diversify the design space, while DL chaos hybrids for wireless/secondary user systems and hash table-augmented chaotic key streams illustrate deployment-specific optimizations. In parallel, DL has been explored for multi-image simultaneous security via interference-coded aperture correlation holography, privacy-preserving captioning with partial encryption, and joint compression encryption of CT images for secure telemedicine.

In contrast to prior deep learning keystream generators such as DeepKeyGen [12], the neural component in our design is strictly assistive: the LSTM predicts short windows of trajectories for a fixed four-dimensional chaotic driver, and a candidate window is admitted as keystream only if its largest Lyapunov exponents are strictly positive on all four coordinates. The cipher itself is fully explicit and invertible, with two scrambling stages followed by rule-driven DNA cross diffusion, and all secret material is classical (chaotic initial states/parameters and DNA rule selections); network weights are never part of the key space. This architectural choice preserves deterministic invertibility and decouples cryptographic keying from model parameters while enforcing dynamical soundness via an LLE gate.

## 2. Related Work

Early and influential studies established DNA operations (e.g., rule-based addition/XOR) as effective diffusion tools within chaos-driven image ciphers [13,14]. Belazi et al. tailored a chaos DNA scheme specifically for medical images, illustrating practicality in clinical settings [15]. To strengthen confusion and enlarge key spaces, designers explored higher-dimensional and multi-wing maps. A new five-dimensional multi-band, multi-wing chaotic system, combined with QR decomposition, improves mixing and resists chosen plaintext attacks [7]. An improved four-dimensional chaotic system, enhanced by evolutionary operators, increases randomness and sensitivity [16]. Traversal and permutation strategies are equally central: Josephus traversing, synchronized with hyperchaotic dynamics, leads to strong scrambling for medical images [6], while Fisher–Yates scrambling, paired with filter diffusion, offers unbiased permutation and robust diffusion [5]. Works focused explicitly on medical constraints include an improved chaos-based cryptosystem for encryption/decryption in clinical workflows [17] and a scheme for securing multiple medical images simultaneously [18]. Region awareness and selectivity have also been explored: encryption using edge maps to preserve diagnostic structures [19], selective protection with DNA cryptography for efficiency [20], and algorithms validated on both grayscale and color medical images [21]. Complementing these, blockchain-driven cloud protection, combined with a chaotic tent map, targets integrity and auditability in distributed environments [8], while a lightweight IoMT-oriented technique addresses resource-constrained deployments [10]. Recent patient privacy-focused designs highlight Josephus scrambling, plus dynamic cross diffusion [4], and ROI-based protection via chaotic S-boxes to prioritize diagnostically critical regions [9]. Hyperchaotic/DNA combinations also extend into healthcare IoT through a novel hyperchaotic map and DNA cubes [11].

Progress continues on map construction and bio-inspired modeling. A novel one-dimensional chaotic system derived from the three-strand structure of DNA offers fresh keystream dynamics for image encryption [22]. Classical attractors and families remain relevant as building blocks and benchmarks; Rössler’s continuous chaos [1] and the Chen-Ueta attractor [2] are examples. Similarly, four-dimensional systems that expand state space for stronger key sensitivity [3] are also relevant. Foundational guidance on the cryptographic requirements for chaos-based systems (key space, sensitivity, and statistical uniformity) remains a touchstone for evaluating these designs [23]. Earlier color image efforts, which mix skew tent maps with hyperchaotic cellular neural networks, exemplify the long-standing drive toward higher complexity [24].

Deep learning now pervades image encryption pipelines in various ways. Key stream learning is illustrated by Deep Key Gen, which educates a stream cipher generator for medical images [12]. Feature based strategies employ CNNs and encoders or neural encoders for controlling DNA encoding or for conducting end to end encryption/decryption: CNN-based DNA encryption [25], tailor-made neural networks with DNA encoding for color images [26], and a deep learning feature encoding/decoding regime acting itself as a cipher [27]. Generative and model-based combination schemes incorporate double encryption through enhanced GANs, complemented with a hyperchaotic system [28] and model learning RBF-aided hyperchaotic schemes [29]. Application-driven work illustrates encryption for primary user/wireless devices through DL with chaos [30], along with DL + chaotic map key streams aided through hash tables [31]. Going beyond a single-image scenario, a number of images can remain securely manageable through deep learning-aided interferenceless coded aperture correlation holography [32]. Privacy–utility trade-offs are investigated through partial encryption in privacy-enhancing image captioning [33], while telemedicine pipelines are aided with joint encryption–compression of CT images using DL for bandwidth–robustness compromise [34]. A recent overview integrates the basics, trends, challenges, and open questions for DL-based image encryption, providing a roadmap for future work [35].

Recent DL-based encryption methods can be grouped as follows: (i) keystream generators that learn a stream cipher (e.g., DeepKeyGen) to drive permutation/diffusion; (ii) feature-encoder ciphers that map images via learned encoders/decoders or CNN-guided DNA rules; (iii) generative hybrids that combine GAN-based transformations with hyperchaotic diffusion; and (iv) model-assisted chaos wherein ML (e.g., RBF nets) tunes or augments chaotic systems. Our approach differs along three axes: (a) deterministic invertibility with explicit cryptographic primitives (two scramblings + DNA diffusion) rather than end-to-end learned transforms; (b) keying rooted in dynamical systems the secret key is defined by 4D chaotic initial states and DNA rule selections while LSTM is used only to predict candidate chaotic traces; and (c) LLE gating where a candidate keystream is admitted if its largest Lyapunov exponents are strictly positive, thus rejecting nonchaotic predictions before use. This gate enforces dynamical validity that typical DL pipelines do not explicitly verify.

## 3. Preliminaries

In this section, we present the key concepts that form the basis for encryption algorithm. We first introduce the Long Short-Term Memory (LSTM) neural network, which is used to predict the chaotic sequences with high accuracy. The proposed scheme used 4D chaotic system, a nonlinear dynamic used to generate the pseudorandom matrices. We use the largest Lyapunov exponent (LLE), which quantifies the system’s sensitivity to its initial condition, to verify the chaotic nature of a 4D chaotic system. Finally, we discuss the DNA diffusion, an encoding scheme which is inspired by the biological system that enhanced the confusion in the image pixels.

### 3.1. Four-Dimensional Chaotic System

Chaos theory examines differential equation systems, where even slight modifications to the original condition can have wildly divergent results, a property commonly known as the Butterfly Effect. These systems are completely deterministic, meaning their initial conditions and system parameters are defined; however, their extreme sensitivity and complex trajectories make them unpredictable. The chaos theory emerged in the mid-20th century, when researchers observed that deterministic models could generate highly irregular and unpredictable behavior. In 1963, when Edward Lorenz studied weather systems, he revealed that small changes in initial velocity or temperature can result in different outcomes, such as storms or clear skies. Following Lorenz’s model, other chaotic systems such as the Rössler system (1976) and Chen system (1999) were introduced, each system discussed the new insights into nonlinear dynamic systems [1,2].

In recent years, the chaos theory has gained attention in the field of cryptography, especially in image encryption due to its properties of sensitivity, pseudorandom matrices, and ergodicity. These properties of chaotic systems allow for generating unpredictable pseudorandom matrices that can be effectively used for pixel scrambling and diffusion in image data. For modern image encryption schemes, higher-dimensional chaotic systems, such as 4D models, are more suitable, unlike traditional random number generators [23]. In this paper, we utilize the four-dimensional chaotic system proposed by the Qi et al. [3], which is mathematically is defined as follows:

(1) $$\left\{\begin{array}{cc} {\dot{x}}_{1} & =a\left(x_{2}-x_{1}\right)+x_{2}\;x_{3}\;x_{4}\;,\\ {\dot{x}}_{2} & =b\left(x_{1}+x_{2}\right)-x_{1}\;x_{3}\;x_{4}\;,\\ {\dot{x}}_{3} & =-c\;x_{3}+x_{1}\;x_{2}\;x_{4}\;,\\ {\dot{x}}_{4} & =-d\;x_{4}+x_{1}\;x_{2}\;x_{3}\;, \end{array}\right.$$

where the $x_{i}\left(t\right)$ represent the state parameter for i=1,…,4, and {a, b, c, d} represent the initial condition of the chaotic system.

**Figure 1:**
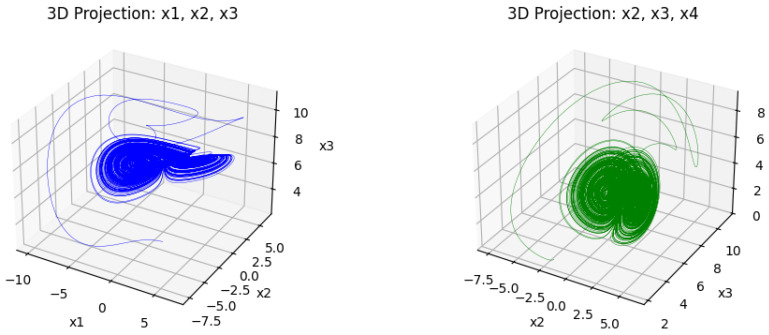
Three-dimensional projection of the 4D chaotic system.

**Figure 2:**
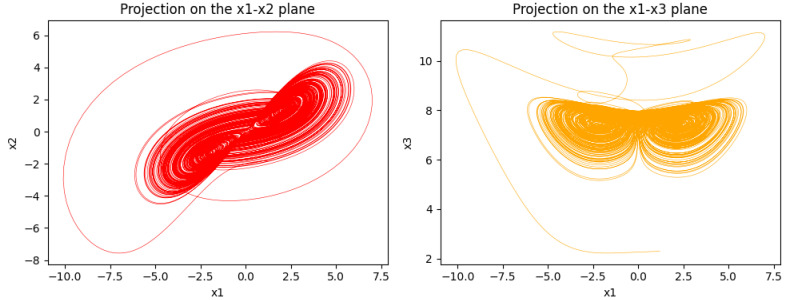
Two-dimensional projection of the 4D chaotic system.

The following Figure 1 and Figure 2 represent the dynamical behavior of the four-dimensional chaotic system through its projection. Figure 1 presents the three-dimensional projection of the chaotic system, where intricate trajectories indicate its nonlinear and aperiodic behavior. These complex attractors show the system’s sensitivity to the initial condition, which makes it suitable for image encryption. Figure 2 presents the corresponding 2D projection, which provides a clear visualization and fulfills the chaotic properties. When combined, these trajectories ensure that the underlying four-dimensional chaotic system possesses the complexity and sensitivity required for secure image encryption.

### 3.2. Long Short-Term Memory (LSTM)

**Figure 3:**
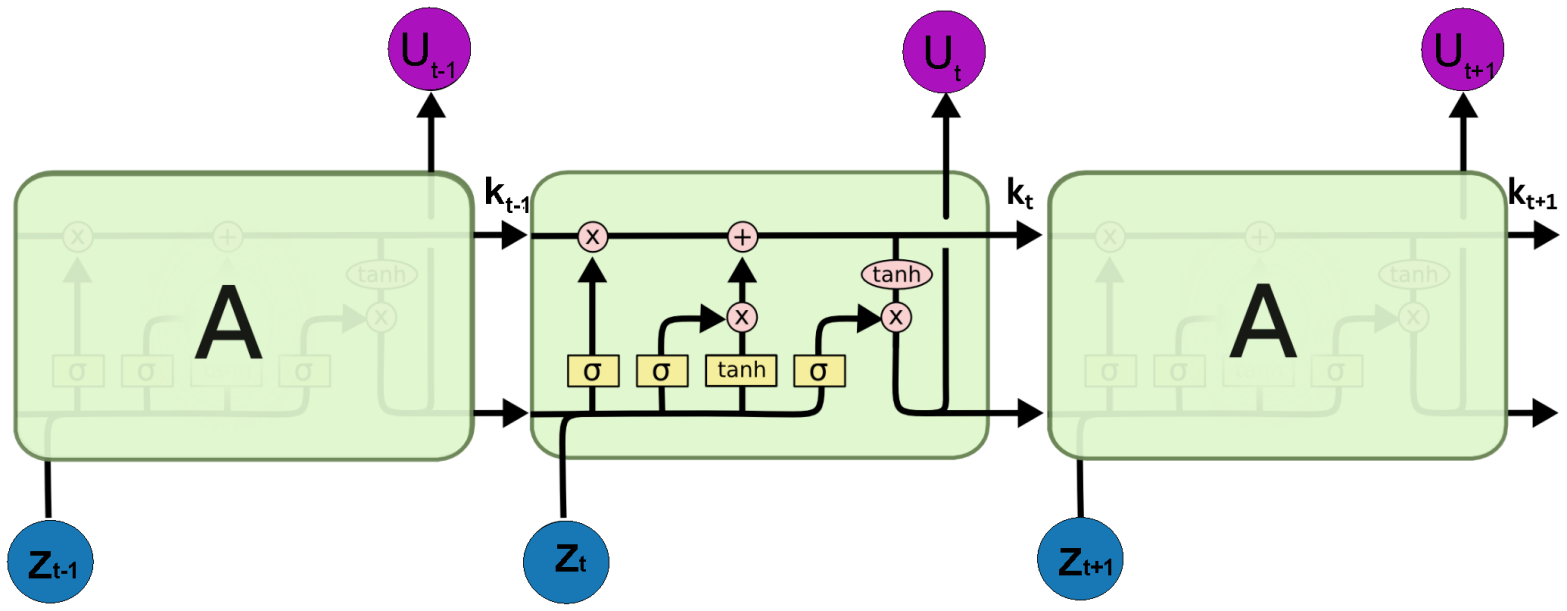
Architecture LSTM network.

Long Short-Term Memory (LSTM) is a specialized type of Recurrent Neural Network, designed to overcome the issue of traditional RNNs in capturing long-term dependencies. LSTMs are better at remembering information over a long period of time. It can selectively add, remove, or update the information by utilizing its gate mechanism, namely input, forget, and output gates [36]. This mechanism makes it effective for modeling time series data and nonlinear dynamics. LSTMs are more suitable for chaotic systems due to their ability to learn patterns and make predictions of chaotic sequences. The LSTM comprises two memory cells: the hidden state memory cell, which is utilized for short-term memory, and the cell state memory cell, which is employed for long-term memory. An architecture LSTM network is shown in Figure 3. The LSTM structure is based on the gates, which are defined as follows:

**Forget Gate:** The crucial element of the LSTM cell state that controls how much data from the prior cell state is kept or discarded is the forget gate. It takes the input vector $z_{t}$ at each time step t, multiplies it by the previous hidden state $u_{t-1}$, and then passes it to the sigmoid activation function, which ranges from 0 to 1. Mathematically, it is defined as

$$\varphi_{t}=\sigma \left(W_{\varphi }\left[z_{t},u_{t-1}\right]+b_{\varphi }\right),$$

where $W_{\varphi }$ is the learnable weighted matrix. The output of this gate would be a vector with values between 0 and 1, where 0 means nothing to remember and 1 means all information is saved.

**Input Gate:** The input gate determines how much of the current input should be stored in the cell state, also known as long-term memory. It is used to combine the current input vector $z_{t}$ at time *t* with the previous hidden state $u_{t-1}$ and pass the result through the activation function. Additionally, the input gate determines which portion of the candidate cell state should be added to the current cell state when the LSTM generates the candidate cell state ${\tilde{C}}_{t}$. Mathematically, it is defined as follows:

$$\iota_{t}=\sigma \left(W_{\iota }\left[z_{t},u_{t-1}\right]+b_{\iota }\right),$$

$${\tilde{C}}_{t}=\tanh\left(W_{\tilde{C}}\left[z_{t},u_{t-1}\right]+b_{\tilde{C}}\right),$$

$$k_{t}=\varphi_{t}⊙k_{t-1}+\iota_{t}⊙{\tilde{C}}_{t},$$

where $W_{\iota }$ and $W_{\tilde{C}}$ are the weighted matrices of input gate and candidate cell state, respectively; $k_{t}$ is the final cell state (long-term memory); and ⊙ is the symbol for the dot product of multiplication between vectors.

**Output Gate:** The output gate determines which information from the update cell state is sent to the output and becomes part of the next hidden state memory. It is defined as follows:

$$\vartheta_{t}=\sigma \left(W_{\vartheta }\left[z_{t},u_{t-1}\right]+b_{\vartheta }\right),$$

$$u_{t}=\vartheta_{t}⊙\tanh\left(k_{t}\right),$$

#### Comparison of Actual and Predicted Chaotic System

**Figure 4:**
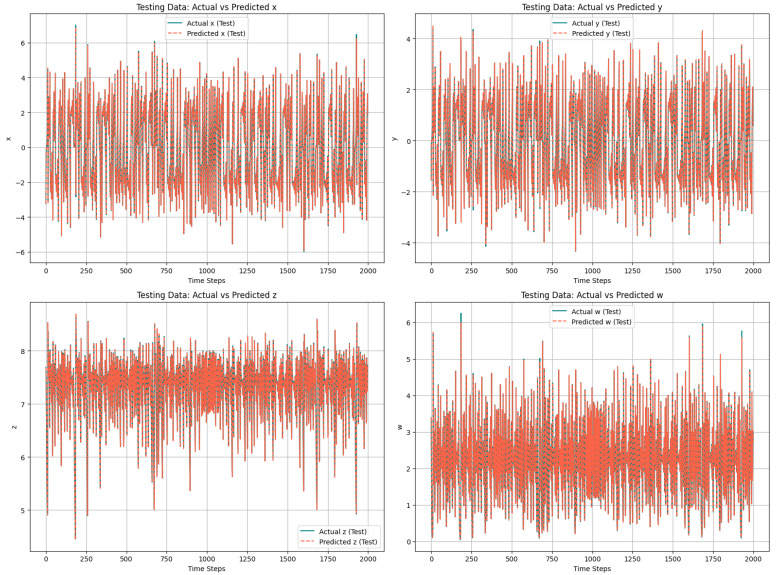
Comparison of actual chaotic data with forecasting of LSTM model.

To evaluate the LSTM model’s efficiency, the predicted chaotic sequence is compared to the actual chaotic sequence obtained from the system’s differential equation. The high degree of overlap between the actual and forecasted values ensure the accuracy of the LSTM model in predicting the chaotic sequences from the trained model. Figure 4 shows this comparison across all the four variables *x*, *y*, *z*, and *w*. Each subplot in the figure shows one dimension of the system. The solid black lines represent the actual chaotic sequence, while the dashed red lines are for the predicted chaotic sequence of the LSTM model. As shown in the following figure, the predicted trajectory closely aligns with the actual chaotic data across all time steps, demonstrating LSTM’s ability to learn the nonlinear temporal dependencies of the chaotic system.

**Figure 5:**
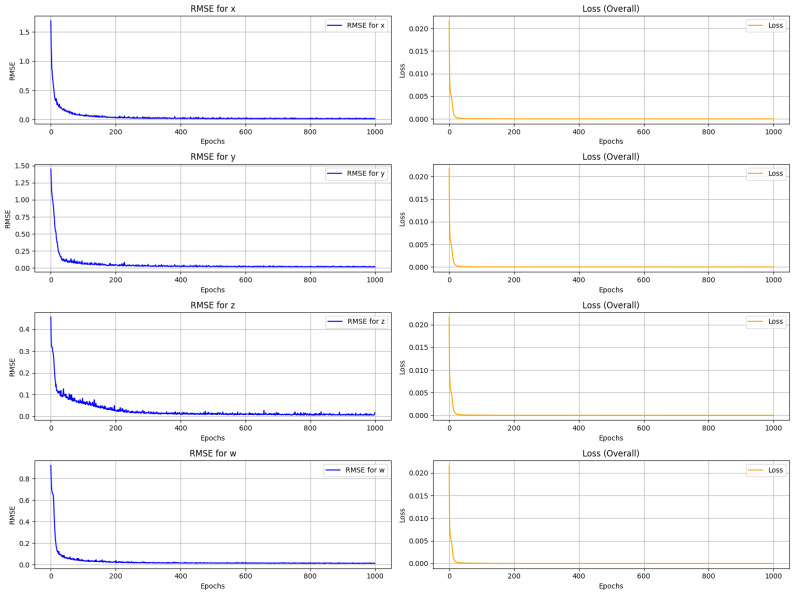
Root mean square error (RMSE) and loss function over all the four variable.

The root mean square error (RMSE) between the actual and expected sequences of the 4D chaotic system is calculated to assess the LSTM model’s expected performance. The low trajectory of RMSE across all the epochs illustrates that the model learned efficiently. The mean square error (MSE), which is the square difference between the actual and predicted values, is used as a loss function during training. As shown in Figure 5, the RMSE is calculated across all four variables of the 4D chaotic system. In all the cases, the trajectory sharply decreases and gradually converges to zero, indicating the progress of the LSTM model training. Similarly, the left column of Figure 5 demonstrates that the MSE drops rapidly and stabilizes close to zero, confirming that the model successfully minimizes the prediction error.

### 3.3. Largest Lyapunov Exponent (LLE)

The largest Lyapunov exponent (LLE) is a key quantitative measure used to identify the nature and behavior of nonlinear dynamical systems. It calculates the average exponential rate of time-dependent divergence between two trajectories in the system’s phase space. If the computed LLE value is positive, it indicates that the nonlinear system is chaotic and sensitive to the initial condition, while the zero and negative values of LLE correspond to the periodic or stable position of the system. In the context of image encryption, the positive LLE ensures the unpredictability and randomness of the chaotic system mentioned, which is essential in cryptographic applications.

**Figure 6:**
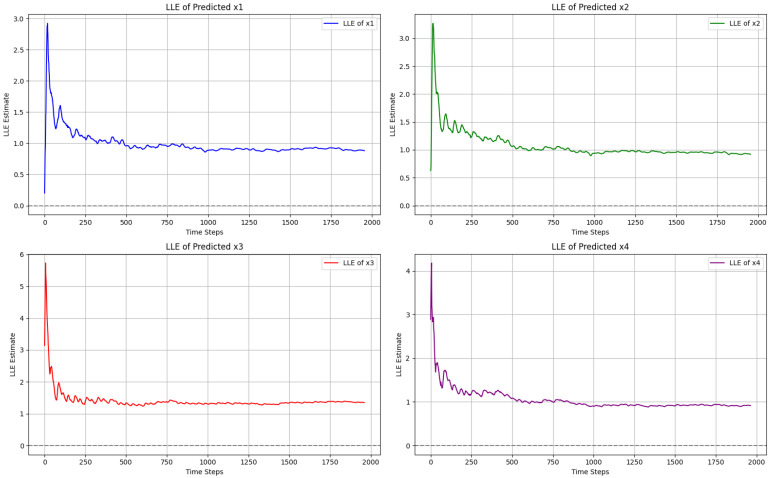
Largest Lyapunov exponent of 4D hyperchaotic system.

As shown in Figure 6, we compute the LLE across all four variables $x_{1}$, $x_{2}$, $x_{3}$, and $x_{4}$ of the proposed 4D chaotic system to check its chaotic nature. For each variable, the obtained curve remains positive, which indicates the system’s sensitivity to the initial condition and, therefore, its chaotic nature. Among these, the stabilized LLE values observed are x1: 0.880917, x2: 0.918967, x3: 1.349992, and x4: 0.914473. All these positive values indicate the complexity of a 4D chaotic system, which is required for image encryption.

### 3.4. Deoxyribonucleic Acid (DNA)

DNA encoding has emerged as a promising technique in image encryption due to its capability to enhance randomness and complexity by applying the principles of DNA encoding. According to the predetermined DNA encoding principles, the color image is first transformed into a binary sequence, which is then mapped to the DNA bases {Adenine: A, Thymine: T, Cytosine: C, Guanine: G}. There are eight different DNA encoding rules employed, where each 2-bit binary string (00, 01, 10, and 11) is mapped to a nucleotide, providing multiple encoding representations in image encryption. Once all the pixel decimal values are encoded, DNA operations such as addition, subtraction, and XOR are applied according to the predefined rules to diffuse the image pixel values and spread small changes across the entire image. As shown in Table 1, the binary string “01” mapped to G by the rule No. 02 and C in the rule No. 07 indicates the power of adding confusion and diffusion in the image encryption.

In image encryption, DNA conversion (DNA_Conv) is used to map binary data to symbolic DNA sequences, and decimal conversion (DEC_Conv) is the reverse process to convert the DNA sequence back to a binary string. For example, consider the pixel value is 201, whose binary string is 11001001. Splitting this string into 2 bit produces 11, 00, 10, and 01. By rule 02, these binary strings map to T, A, C, and G, which corresponds to the DNA sequence TACG. Symbolically, it is defined as DNA_Conv(201, 2) = TACG. We can also restore the pixel value to decimal by following the same backward steps from the DNA sequence to decimal, which is written as Dec_Conv(TACG,2)=201.

DNA operations are key to DNA-based encryption, as they enable the direct manipulation of DNA sequences rather than binary strings. There are three main operations of DNA addition (+), subtraction (−), and XOR (⊕), which are defined over nucleotides rather than integers. For example, to apply the addition operation on DNA sequences ATCG and TCAG by the rule 01, we write it as +(ATCG,TCGA,1)=CGCT. Similarly for the substraction and XOR operations, −(ATCG,TCGA,1)=GGCA and ⊕(ATCG,TCGA,1)=TACA, respectively; see Table 2.

## 4. The Proposed Encryption Method

**Figure 7:**
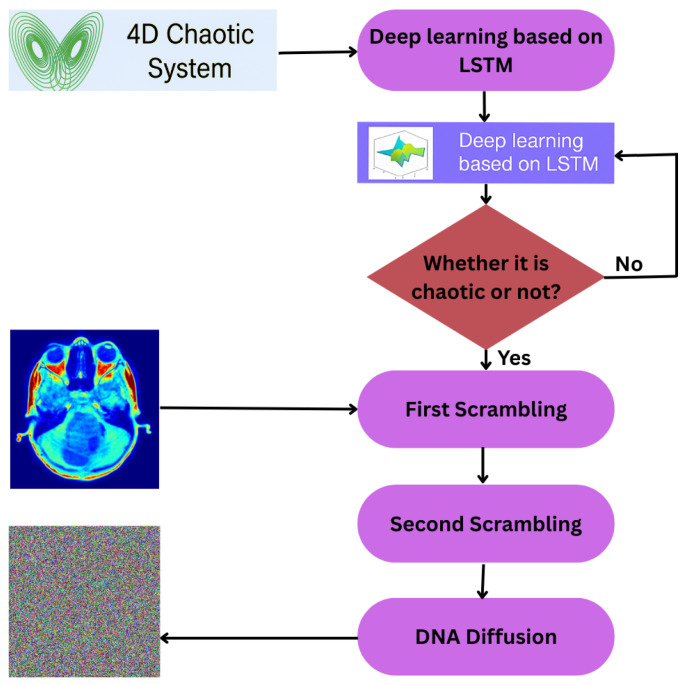
Flowchart of encryption algorithm.

To ensure robust randomness in the proposed image encryption, we first use a medical image as input and employ the LSTM model’s predicted sequences for 4D chaotic systems to generate pseudorandom matrices. The image is then scrambled by shuffling its pixels, followed by DNA encoding and operations, which are controlled by pseudorandom matrices. Finally, DNA diffusion is applied, which spreads even a minor change across the entire image, ensuring a high level of randomness and sensitivity, and secure medical images. Figure 7 shows the complete flowchart of the algorithm.

### 4.1. Preprocessing of Image Encryption

This step ensures that the medical image is ready for encryption by resizing, normalizing, and separating it into the RGB channels. We take a color image P(M×N) as an input and produce the output of an encrypted image C(M×N) by applying the well-designed encryption algorithm. Here, M and N are the rows and columns, respectively.

- Input: Medical color image P of M×N size.
- Output: The encrypted image ϕ of same size as P.
- Initial Key: A set of initial key values $K=\{x_{0},y_{0},z_{0},w_{0}\}$ for initializing the 4D chaotic system.

### 4.2. Generation of Pseudorandom Matrices

1. Initializing Chaotic System: We have the four-dimensional chaotic system which will be initialized with the initial key K. Next, we generate the chaotic sequences $\left\{x_{1\upsilon }\right\}$, $\left\{x_{2\upsilon }\right\}$, $\left\{x_{3\upsilon }\right\}$, and $\left\{x_{4\upsilon }\right\}$ by applying the numerical solver method, where υ=1,2,…,n and n>MN.
2. LSTM Predictions: By using these chaotic sequences $\left\{x_{1\upsilon }\right\}$, $\left\{x_{2\upsilon }\right\}$, $\left\{x_{3\upsilon }\right\}$, and $\left\{x_{4\upsilon }\right\}$ we train our LSTM model. With a trained model, we predict the new chaotic sequences $\left\{x_{1\upsilon }^{\prime }\right\}$, $\left\{x_{2\upsilon }^{\prime }\right\}$, $\left\{x_{3\upsilon }^{\prime }\right\}$, and $\left\{x_{4\upsilon }^{\prime }\right\}$. If one of the chaotic sequences is not chaotic, we pass it back to the LSTM to make new predictions.
3. Chaos Verification: To ensure that the predicted chaotic sequences $\left\{x_{1\upsilon }^{\prime }\right\}$, $\left\{x_{2\upsilon }^{\prime }\right\}$, $\left\{x_{3\upsilon }^{\prime }\right\}$, and $\left\{x_{4\upsilon }^{\prime }\right\}$ exhibit the truely chaotic behavior, we apply the largest Lyapunov exponent on the predicted chaotic sequences. If one of the chaotic sequences is not chaotic. We pass it back to the LSTM to make new predictions.
4. Construction of Pseudorandom Matrices: Next, we generate the four pseudorandom sequences $A_{1}$, $A_{2}$, $A_{3}$, and $A_{4}$ by using the predicted values $\left\{x_{1\upsilon }^{\prime }\right\}$, $\left\{x_{2\upsilon }^{\prime }\right\}$, $\left\{x_{3\upsilon }^{\prime }\right\}$, and $\left\{x_{4\upsilon }^{\prime }\right\}$ as follows:
  $$\begin{array}{cc} A_{1}\left(\upsilon \right) & =\left\lfloor (\right|x_{1\upsilon }^{\prime }\left|\;\mathrm{mod}\;1\right)\cdot 10^{14}\rfloor \;\mathrm{mod}\;256.\\ A_{2}\left(\upsilon \right) & =\left\lfloor (\right|x_{2\upsilon }^{\prime }\left|\;\mathrm{mod}\;1\right)\cdot 10^{14}\rfloor \;\mathrm{mod}\;256.\\ A_{3}\left(\upsilon \right) & =\left\lfloor (\right|x_{3\upsilon }^{\prime }\left|\;\mathrm{mod}\;1\right)\cdot 10^{14}\rfloor \;\mathrm{mod}\;256.\\ A_{4}\left(\upsilon \right) & =\left\lfloor (\right|x_{4\upsilon }^{\prime }\left|\;\mathrm{mod}\;1\right)\cdot 10^{14}\rfloor \;\mathrm{mod}\;256. \end{array}$$
  where υ=1,2,…,M·N.

### 4.3. First Scrambling Scheme

The act of rearranging and shuffling the pixel values from their original positions to fully distort the visual structure of the image is known as scrambling in image encryption. Similarly, we choose the Fisher–Yates and Thorp’s method, which is a very powerful technique for adding randomness in the positions of the image pixels. For this scrambling, we must follow the following steps to obtain the scrambled image.

1. First, we will generate the random sequence J of binary numbers {0, 1} of the size 1×MN and keep it saved for decryption purposes.
2. Take the original image P, and convert it to the one-dimensional array as follows:
  P=reshape(P,1,M×N).
3. If the ith position of J sequence is 1, then swap P(i) and P(j), where j is computed as follows:
  $$j=\left\lfloor \frac{i+1}{{\left(MN-1\right)}^{3}}\times 10^{16}\right\rfloor \;\mathrm{mod}\;MN.$$
4. If the ith position of J is 0, then rotate P(i) to P(j), P(j) to P(w), and P(w) to P(i), where w is computed as follows:
  $$w=\left(i+j+\left\lfloor \frac{MN}{2}\right\rfloor \right)\;\mathrm{mod}\;MN.$$
5. For the decryption purpose, we have to keep saving these operations (swap and rotate) corresponding to each pixel value in P. Reshape P back to the size M×N as follows:
  A=reshape(P,M,N),
  where *A* is the final scrambled image.

### 4.4. Second Scrambling Process

**Figure 8:**
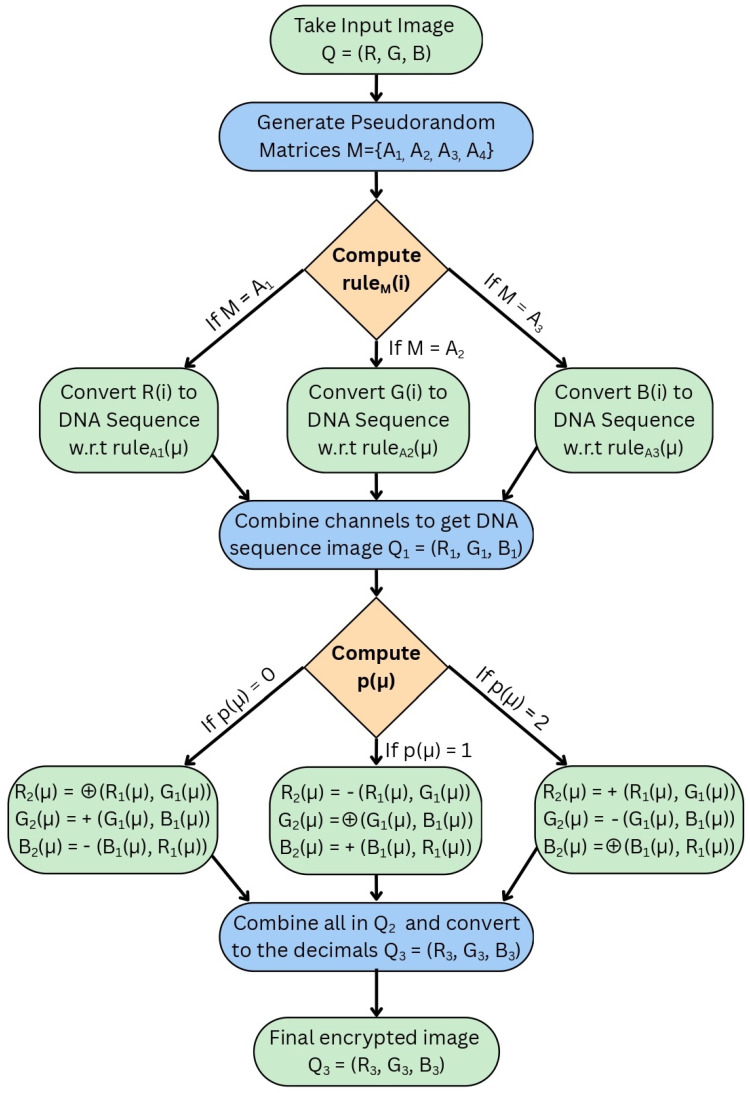
DNA diffusion flowchart.

Now we apply the second scrambling scheme after the initial permutation to further add randomness in the image pixels. This additional layer of scrambling enhances complexity by shuffling the order. This scrambling ensures that any residual pixels pattern is removed from the first scrambling. Importantly, the confused image will make it more difficult for an attacker to extract the structural information. Its flowchart is shown in Figure 8.

1. Take the first scrambled image A, and split it into an equal number of sub-images with size $h=2^{n}$, where n = {2, 3, 4}.
2. Divided every sub-image further into blocks of size $h=2^{l}$, where l∈{2,3,…,n}, and save these random generated values for each sub-image into R, with index $i=1:\left(\frac{MN}{h^{2}}\right)$.
3. Next, apply the zig-zag pattern to each sub-image and its blocks. After this, rotate them 90 degrees counterclockwise.
4. Generate the random permutation r with $\frac{MN}{h^{2}}$, rearrange the sub-images with respect to r positions. Finally, Q(R,G,B) is a scrambled image.

### 4.5. DNA Diffusion

DNA diffusion plays a key rule in the image encryption that provides us with a strong level of modification in the image pixels and ensures high security. In this method, we first convert the image pixels to DNA sequences and then apply DNA operations, such as addition, subtraction, and XOR. These operations modify pixel values using pseudorandom matrices and produce highly unpredictable output. Because of this, even a slight alteration to the original pixel value results in a completely new encrypted image, making it extremely sensitive and ensuring robust defense against differential attacks.

DNA Conversion: For the DNA conversion, we take a scrambled image as an input Q=(R,G,B) with three RGB channels of size M×N, and convert them into a one-dimensional array by inserting one after the other. Next, we use the generated pseudorandom matrices $A_{1}$, $A_{2}$, and $A_{3}$, and get $Q_{1}=\left(R_{1},G_{1},B_{1}\right)$ as follows:
  (2) $$\begin{array}{cc} R_{1}\left(\mu \right) & =DNA\_Conv\left(R\left(\mu \right),\;rule_{A_{1}}\left(\mu \right)\right),\;\\ G_{1}\left(\mu \right) & =DNA\_Conv\left(G\left(\mu \right),\;rule_{A_{2}}\left(\mu \right)\right),\;\\ B_{1}\left(\mu \right) & =DNA\_Conv\left(B\left(\mu \right),\;rule_{A_{3}}\left(\mu \right)\right). \end{array}$$
  where
  (3) $$rule_{M}\left(\mu \right)=\;\mathrm{mod}\;\left(M(\mu ),8\right)+1$$
  for μ=0,1,…,MN−1 and $M=\{A_{1},A_{2},A_{3}\}$. By combining these three channels, we get $Q_{1}=\left(R_{1},G_{1},B_{1}\right)$.
  DNA Diffusion: Next, we apply the DNA operations on the input image $Q_{1}=\left(R_{1},G_{1},B_{1}\right)$ with the help of $A_{4}$ and follow the DNA encoding rules according to Table 1 as follows:
  (4) $$\begin{array}{cc} \mathrm{if}\;p(\mu )=0: & \left\{\begin{array}{c} R_{2}\left(\mu \right)=⊕\left(R_{1}\left(\mu \right),G_{1}\left(\mu \right),rule_{A_{4}}\left(\mu \right)\right),\;\\ G_{2}\left(\mu \right)=+\left(G_{1}\left(\mu \right),B_{1}\left(\mu \right),rule_{A_{4}}\left(\mu \right)\right),\;\\ B_{2}\left(\mu \right)=-\left(B_{1}\left(\mu \right),R_{2}\left(\mu \right),rule_{A_{4}}\left(\mu \right)\right), \end{array}\right. \end{array}$$
  (5) $$\begin{array}{cc} \mathrm{if}\;p(\mu )=1: & \left\{\begin{array}{c} R_{2}\left(\mu \right)=-\left(R_{1}\left(\mu \right),G_{1}\left(\mu \right),rule_{A_{4}}\left(\mu \right)\right),\;\\ G_{2}\left(\mu \right)=⊕\left(G_{1}\left(\mu \right),B_{1}\left(\mu \right),rule_{A_{4}}\left(\mu \right)\right),\;\\ B_{2}\left(\mu \right)=+\left(B_{1}\left(\mu \right),R_{2}\left(\mu \right),rule_{A_{4}}\left(\mu \right)\right), \end{array}\right. \end{array}$$
  (6) $$\begin{array}{cc} \mathrm{if}\;p(\mu )=2: & \left\{\begin{array}{c} R_{2}\left(\mu \right)=+\left(R_{1}\left(\mu \right),G_{1}\left(\mu \right),rule_{A_{4}}\left(\mu \right)\right),\;\\ G_{2}\left(\mu \right)=-\left(G_{1}\left(\mu \right),B_{1}\left(\mu \right),rule_{A_{4}}\left(\mu \right)\right),\;\\ B_{2}\left(\mu \right)=⊕\left(B_{1}\left(\mu \right),R_{2}\left(\mu \right),rule_{A_{4}}\left(\mu \right)\right), \end{array}\right. \end{array}$$
  where
  (7) $$p\left(\mu \right)=\;\mathrm{mod}\;\left(A_{4}\left(\mu \right),3\right),$$
  and
  (8) $$rule_{A_{4}}\left(\mu \right)=\;\mathrm{mod}\;\left(A_{4}\left(\mu \right),8\right)+1,$$
  for μ=0,1,…,MN−1. Now, we have the diffused image $Q_{2}=\left(R_{2},G_{2},B_{2}\right)$ with DNA sequence entries.
  Decimal Conversion: Finally, we convert the DNA sequence of $Q_{2}=\left(R_{2},G_{2},B_{2}\right)$ to the decimal for its RGB channels into a color image. For this, we follow the following steps:
  (9) $$\begin{array}{cc} R_{3}\left(\mu \right) & =DEC\_Conv\left(R\left(\mu \right),\;rule_{A_{1}}\left(\mu \right)\right),\;\\ G_{3}\left(\mu \right) & =DEC\_Conv\left(G\left(\mu \right),\;rule_{A_{2}}\left(\mu \right)\right),\;\\ B_{3}\left(\mu \right) & =DEC\_Conv\left(B\left(\mu \right),\;rule_{A_{3}}\left(\mu \right)\right). \end{array}$$
  where
  (10) $$\begin{array}{c} rule_{A_{1}}\left(\mu \right)=\;\mathrm{mod}\;\left(\mu +R_{3}\left(\mu -1\right),8\right)+1,\;\\ rule_{A_{2}}\left(\mu \right)=\;\mathrm{mod}\;\left(\mu +G_{3}\left(\mu -1\right),8\right)+1,\;\\ rule_{A_{3}}\left(\mu \right)=\;\mathrm{mod}\;\left(\mu +B_{3}\left(\mu -1\right),8\right)+1, \end{array}$$
  for μ=0,1,…,MN−1 and we initialize this iteration with predefined values of $R_{3}\left(-1\right)$, $G_{3}\left(-1\right)$, and $B_{3}\left(-1\right)$. Finally, we have the encrypted image $Q_{3}=\left(R_{3},G_{3},B_{3}\right)$.

### 4.6. Inverse DNA Diffusion

To recover the original medical image from the encrypted data, the decryption process performs the inverse operations of the encryption. The decryption process consists of three main steps: inverse decimal conversion, inverse DNA diffusion, and inverse DNA conversion.

Inverse Decimal Conversion: First, we convert the encrypted image $Q_{3}=\left(R_{3},G_{3},B_{3}\right)$ from decimal to DNA sequences using the same pseudorandom rules $rule_{A_{1}}$, $rule_{A_{2}}$, and $rule_{A_{3}}$ and the same predefined values $R_{3}\left(-1\right),G_{3}\left(-1\right),B_{3}\left(-1\right)$ that are available. Thus, we get the intermediate sequences $Q_{2}=\left(R_{2},G_{2},B_{2}\right)$:
  (11) $$\begin{array}{cc} R_{2}\left(\mu \right) & =DNA\_Conv\left(R_{3}\left(\mu \right),\;rule_{A_{1}}\left(\mu \right)\right),\\ G_{2}\left(\mu \right) & =DNA\_Conv\left(G_{3}\left(\mu \right),\;rule_{A_{2}}\left(\mu \right)\right),\\ B_{2}\left(\mu \right) & =DNA\_Conv\left(B_{3}\left(\mu \right),\;rule_{A_{3}}\left(\mu \right)\right). \end{array}$$
  Inverse DNA Diffusion: Next, we recover the sequences $Q_{1}=\left(R_{1},G_{1},B_{1}\right)$ by applying the inverse of the DNA diffusion where we used the addition, substraction, and XOR operations with the $rule_{A_{4}}\left(\mu \right)$; here, we apply the reverse operations depending on
  (12) $$p\left(\mu \right)=\;\mathrm{mod}\;\left(A_{4}\left(\mu \right),3\right),\;rule_{A_{4}}\left(\mu \right)=\;\mathrm{mod}\;\left(A_{4}\left(\mu \right),8\right)+1.\;$$
  The inverse relations are as follows:
  (13) $$\begin{array}{cc} \mathrm{if}\;p(\mu )=0: & \left\{\begin{array}{c} B_{1}\left(\mu \right)=+\left(B_{2}\left(\mu \right),R_{2}\left(\mu \right),rule_{A_{4}}\left(\mu \right)\right),\\ G_{1}\left(\mu \right)=-\left(G_{2}\left(\mu \right),B_{1}\left(\mu \right),rule_{A_{4}}\left(\mu \right)\right),\\ R_{1}\left(\mu \right)=⊕\left(R_{2}\left(\mu \right),G_{1}\left(\mu \right),rule_{A_{4}}\left(\mu \right)\right), \end{array}\right. \end{array}$$
  (14) $$\begin{array}{cc} \mathrm{if}\;p(\mu )=1: & \left\{\begin{array}{c} B_{1}\left(\mu \right)=-\left(B_{2}\left(\mu \right),R_{2}\left(\mu \right),rule_{A_{4}}\left(\mu \right)\right),\\ G_{1}\left(\mu \right)=⊕\left(G_{2}\left(\mu \right),B_{1}\left(\mu \right),rule_{A_{4}}\left(\mu \right)\right),\\ R_{1}\left(\mu \right)=+\left(R_{2}\left(\mu \right),G_{1}\left(\mu \right),rule_{A_{4}}\left(\mu \right)\right), \end{array}\right. \end{array}$$
  (15) $$\begin{array}{cc} \mathrm{if}\;p(\mu )=2: & \left\{\begin{array}{c} B_{1}\left(\mu \right)=⊕\left(B_{2}\left(\mu \right),R_{2}\left(\mu \right),rule_{A_{4}}\left(\mu \right)\right),\\ G_{1}\left(\mu \right)=+\left(G_{2}\left(\mu \right),B_{1}\left(\mu \right),rule_{A_{4}}\left(\mu \right)\right),\\ R_{1}\left(\mu \right)=-\left(R_{2}\left(\mu \right),G_{1}\left(\mu \right),rule_{A_{4}}\left(\mu \right)\right). \end{array}\right. \end{array}$$
  Inverse DNA Conversion: Finally, we convert the DNA sequences $Q_{1}=\left(R_{1},G_{1},B_{1}\right)$ back to the pixel domain by applying the inverse DNA mapping rules:
  (16) $$\begin{array}{cc} R(\mu ) & =DEC\_Conv\left(R_{1}\left(\mu \right),\;rule_{A_{1}}\left(\mu \right)\right),\;\\ G(\mu ) & =DEC\_Conv\left(G_{1}\left(\mu \right),\;rule_{A_{2}}\left(\mu \right)\right),\;\\ B(\mu ) & =DEC\_Conv\left(B_{1}\left(\mu \right),\;rule_{A_{3}}\left(\mu \right)\right). \end{array}$$
  Thus, we obtain the recovered scrambled image Q=(R,G,B).

### 4.7. Descrambling of Diffusion

1. Second reverse scrambling: We obtained the recovered scrambled image by applying the inverse of DNA diffusion, and the conversion of the final step is reverse scrambling. In this step, the pixel positions are rearranged to their original locations using the same permutation indices that were employed during the encryption process.
  (a) Convert the one-dimensional sequence Q=(R,G,B) to the M×N for each RGB channels. Next, divide Q into sub-images using the $h=2^{n}$, where *n* should be as for scrambling.
  (b) Using vector *r*, return the sub-images to their original position.
  (c) Furthermore, divide the sub-images into blocks of the same size using vector *R*.
  (d) At last, apply the reverse rotation of 90 degrees and reverse zig-zag operation to each block, resulting in a scrambled image A.
2. First reverse scrambling: For the efficient performance of the descrambling process, we assume the availability of a complete operations sequence and binary sequence J, applied during the scrambling. To completely reverse the scrambling procedure, we have to start from the last operation and proceed it backward.
  (a) According to the corresponding value in the binary sequence J from the end to the beginning. If J(i) = 1, then retrieve the value of i and j from the saved operation of $A^{\prime }$ and simply swap them back to their position.
  (b) If J(i) = 0, then get back the values of i, j, and w from the save operations. During scrambling, the values were rotated forward, but now we need to rotate them backward by introducing a temporary variable in Python 3.10.
    $$\mathrm{temp}\leftarrow x_{i},$$
    $$x_{i}\leftarrow x_{w},$$
    $$x_{w}\leftarrow x_{j},$$
    $$x_{j}\leftarrow \mathrm{temp},$$
    where $x_{i}$ represents the value at position *i*.
  (c) Continue until all the operations are reversed. Finally, reshape the 1D array $A^{\prime }$ back to its original image dimensions to obtain the descrambled image *A*.

**Figure 9:**
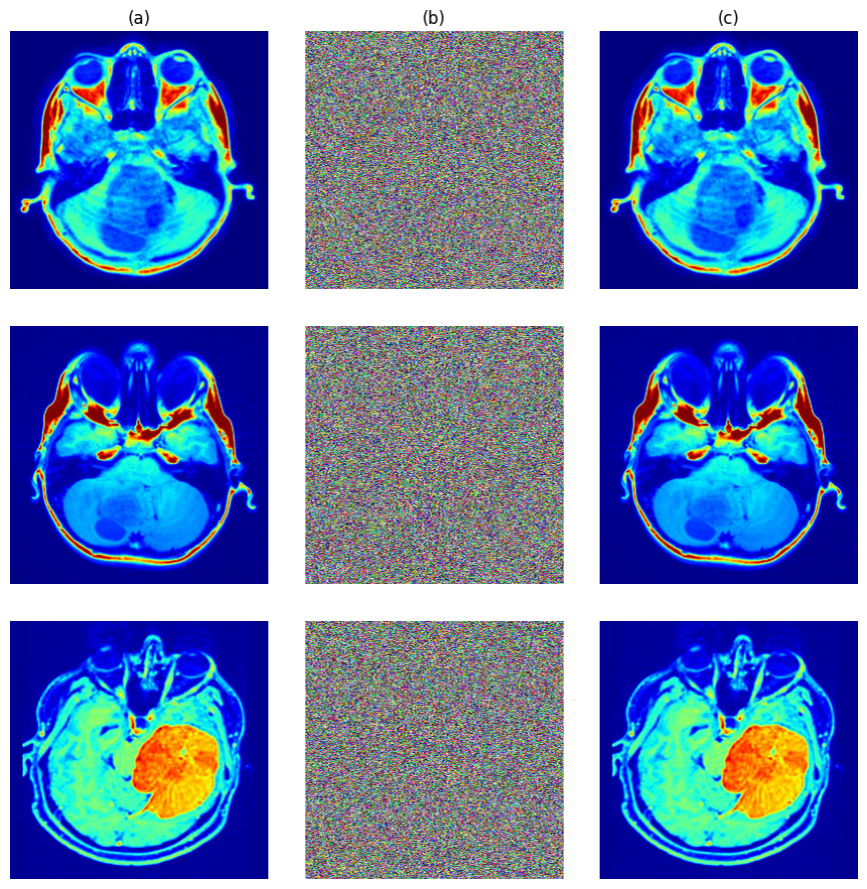
(**a**) Original. (**b**) Encrypted. (**c**) Decrypted images.

The test images in Figure 9a went through the entire encryption process and provided encrypted outputs with matching corresponding decryptions. One can see from Figure 9b that encryption fully masked all the visual features in the originals while ensuring visual confidentiality. With rightful secret keys, successful restoration of originals was performed upon decryption, as shown in Figure 9c, exhibiting both preciseness and reversibility.

## 5. Security Analysis

We evaluated the security of the suggested encryption algorithm against a range of traditional attacks, including differential, statistical, and brute-force attacks. According to statistical research, the encrypted image’s uniform histogram distribution and low correlation between neighboring pixel values make it challenging for a hacker to obtain any valuable data. Additionally, the sensitivity of even slight modifications to the encryption key and the initial plain image produces completely distinct outcomes.

### 5.1. Key Space Analysis

The entire number of keys that can be created and utilized during the encryption method is verified using key space analysis. If your encryption scheme is sufficiently large, which is crucial for resisting brute-force attacks, since an attacker needs to try every possible key to break the encryption scheme, it is secure. An encryption algorithm is considered to be secure if its key space is greater than $2^{100}$, and in practice, a key space greater than $2^{128}$ is considered computationally infeasible to exhaust using modern computation resources. Therefore, a larger and more diverse key space has stronger resistance against the brute-force attacks.

In the proposed image encryption, the secret key consists of four initial condition components a,b,c,d; chaotic system parameters $x_{0},y_{0},z_{0},w_{0}$; and the selection of DNA encoding or decoding rules. These eight components of the chaotic system are represented with the floating-point precision of $10^{-14}$, which contribute approximately $10^{14\times 8}=10^{112}\approx 2^{372}$ to the key space. Furthermore, we selected four DNA rules for the encryption process, each of which was chosen from the eight different rules, adding another factor of $8^{4}=2^{12}$. As a result, the suggested encryption scheme’s total key space is approximately $2^{384},$ which is significantly larger than the typical key space and provides a robust defense against brute-force attacks; see Table 3 for comparison.

### 5.2. Entropy Analysis

The statistical examination of measuring randomness in encrypted images, which reveals uncertainty in pixel values, is called entropy analysis. In image encryption, it is used to evaluate the probability for each pixel to be unpredictable. The optimal entropy value for an 8-bit image is 8, indicating that every pixel value has an equal chance of appearing. If the entropy value for an encryption scheme is close to 8, it indicates that the encryption algorithm produces results with high randomness and minimal patterns, which makes it difficult for an attacker to predict the pixel value and extract any meaningful information. Therefore, a higher entropy value indicates the strength and security of a medical image encryption algorithm. It is calculated as follows:

$$I\left(s\right)=\sum_{\iota =0}^{2^{n}-1}\gamma \left(\iota \right)\log_{2}\frac{1}{\gamma (\iota )},$$

where I(s) represent the Shannon’s entropy, and γ(ι) represents the probability that pixel ι will be present in the encrypted picture.

Table 4 lists the outcomes of the entropy analysis for several medical encrypted photos. The entropy values for the red, green, and blue channels are presented, which are consistently close to the ideal value of 8, with average entropy values of 7.9944, 7.9977, and 7.9959, respectively. These high entropy values ensure that the proposed encryption algorithm produces encrypted images that are completely random, with all the pixel values uniformly distributed. See Table 5 for entropy comparison.

### 5.3. Correlation Coefficient

This analysis determines the direction and intensity of a linear relationship between two variables. Its range is from −1 to 1, where 1 indicates a strong correlation between two variables (one increases, the other also increases), and −1 indicates the negative correlation between two variables (as one increases, the other decreases), and a value of correlation coefficient near 0 indicates that there is no relation between the variables. The correlation coefficient is used in image encryption to quantify the degree of correlation between neighboring pixels. Adjacent horizontal, vertical, and diagonal pixels in the original plain image exhibit a high degree of correlation, indicating that their values are fairly close to one another. However, a secure and efficient image encryption algorithm can destroy this correlation so that adjacent pixels always appear random. Mathematically, the correlation coefficient is calculated as follows:

(17) $$\eta =\frac{\mathrm{Cov}(\alpha ,\beta )}{\sqrt{W(\alpha )}\sqrt{W(\beta )}},$$

where,

(18) $$\mathrm{Cov}\left(\alpha ,\beta \right)=\frac{1}{N}\sum_{\ell =1}^{N}\left(\alpha_{\ell }-\mu_{\alpha }\right)\left(\beta_{\ell }-\mu_{\beta }\right),$$

(19) $$W\left(\alpha \right)=\frac{1}{N}\sum_{\ell =1}^{N}{\left(x_{\ell }-\mu_{\alpha }\right)}^{2},$$

(20) $$\mu_{\alpha }=\frac{1}{N}\sum_{\ell =1}^{N}\alpha_{\ell }.$$

Here, α and β are two adjacent pixels in the horizontal, vertical, and diagonal directions, and *N* is the total number of pixels selected from the image.

**Figure 10:**
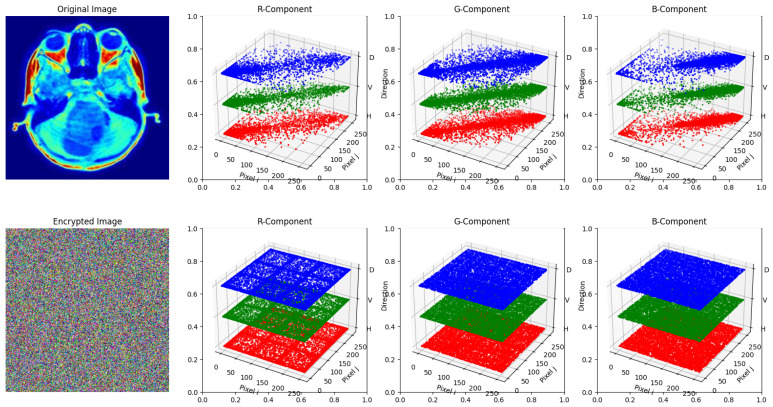
Adjacent pixel correlation distribution in the original and encrypted images.

Figure 10 shows the correlation coefficient for both the original and encrypted images. As seen, the correlation coefficient for the original image across RGB channels remains among the neighborhood pixel values. However, after the encryption, the correlation coefficient across all the RGB channels is uniformly scattered. As shown in Table 6, the correlation coefficient for the original and encrypted images is presented, indicating the correlation between adjacent pixel values. In the original image, the horizontal, vertical, and diagonal pixel correlations are close to 1 (high correlation), indicating that adjacent pixels are strongly related. On the other hand, in the encrypted image, correlation coefficient values drastically drop to 0, from either the positive or negative side, indicating that the relation between neighboring pixels is completely removed.

See Table 7 for the correlation analysis comparing the proposed method with existing schemes.

### 5.4. Histogram Analysis

The histogram analysis plays an important role in evaluating the security strength of the proposed image encryption scheme. The histogram is used to represent the distribution of pixel values, which range from 0 to 255. For the plain color image, the histogram contains the visible pattern that corresponds to the structure and content of the image. However, in the encrypted image, the histogram should be flat and all the pixel values should be distributed evenly without any peaks or visible patterns. This uniformity in the image pixels indicates that the encryption algorithm has successfully randomized the pixel values, making it stronger against statistical attacks.

**Figure 11:**
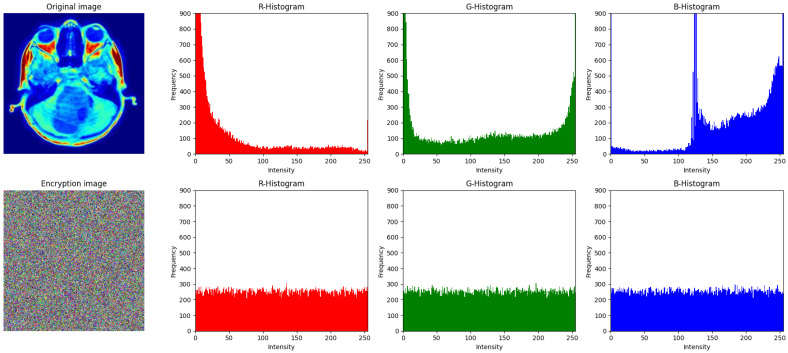
Histogram of plain images and cipher images: (top row) histograms of original image; (bottom row) histograms of cipher image.

The histogram analysis for both the original and encrypted image is displayed in Figure 11. The histograms of the R, G, and B channels in the original image indicate an unequal distribution, with apparent peaks and a discernible pattern in the pixel values. For the encrypted image, the histogram is completely distributed across all channels, with no visible pattern or correlations. This uniformity in the pixel values ensures that the proposed encryption algorithm successfully conceals the statistical information of the original image, making it resistant to various attacks.

### 5.5. Robustness Analysis

**Figure 12:**
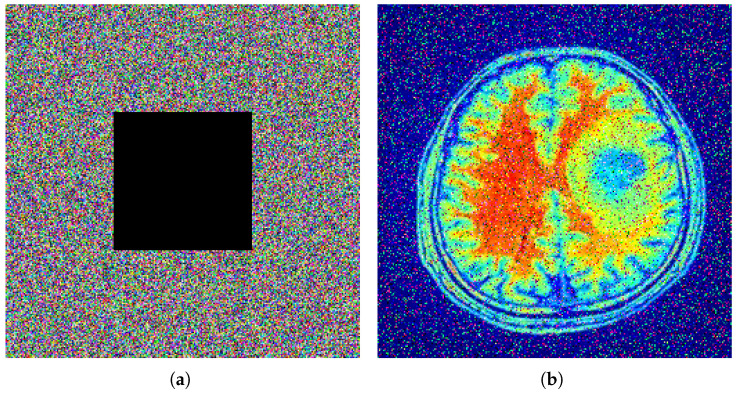

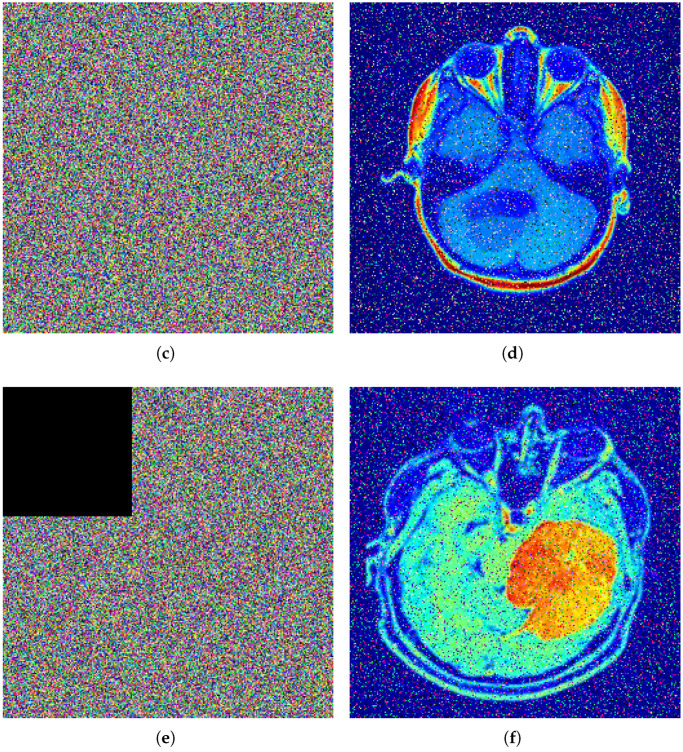
Robustness analysis. (**a**) Encrypted image with a 100×100 black patch missing from the center; (**b**) Decrypted image corresponding to (**a**), showing preserved structural details; (**c**) Encrypted image with added Gaussian noise of variance 0.01; (**d**) Decrypted image corresponding to (**c**), retaining acceptable visual quality; (**e**) Encrypted image with a 100×100 black patch missing from the top-left corner; (**f**) Decrypted image corresponding to (**e**).

This analysis is used to evaluate the resistance against the various intentional or unintentional attacks on the encrypted image. During data storage and transmission, we face various types of data loss, such as corruption, compression, or any other type of damage. Even with such a disturbance, a robust image encryption scheme will always be able to preserve most of the image data, and the decryption process still gives an acceptable visual quality. Figure 12 illustrates the robustness analysis of the proposed encryption algorithm under different types of attacks. In part (a), a 100×100 size of black patch is placed at the center of the encrypted image, but the decryption results still have a recognizable visual pattern. In part (b), Gaussian noise with a variance of 0.01 is applied to the encryption algorithm, and decryption continues to produce the clear, recognizable original image. In part (c), the same 100×100 patch is applied to the top left of the encrypted image, and the decryption process again preserves most of its structure. All of these results mention that the encryption process is vulnerable to data loss and noise interference.

### 5.6. Differential Attack Analysis

Differential attack analysis involves making small modifications to the input image to evaluate the sensitivity of the encrypted image. In this analysis, an attacker changes the value of just one pixel in the original plain image and then checks how much the encrypted image changes as a result. By making a relatively slight alteration to the plain-text image, a robust and safe encryption technique can produce a significant and unpredictable change in the encrypted image. The resistance to different types of differential attacks is measured using two commonly used metrics: UACI (Unified Average Changing Intensity) and NPCR (Number of Pixels Change Rates). NPCR calculates the percentage of pixel values that change in the encrypted image when one pixel value from the original image is altered and then encrypted, whereas UACI calculates the average intensity difference between the two encrypted images. If NPCR and UACI are near 100% and 33%, respectively, the method is deemed strong and secure for picture encryption.

Table 8 displays the average values of the NPCR and UACI tests we performed on the different encrypted images in the red, green, and blue channels. The fact that the NPCR values are nearly 99.6% across all the RGB channels indicates that even a small alteration to the source image can result in a significantly different outcome (cipher image). Likewise, the UACI values, which indicate the average intensity difference between the original and encrypted images, are approximately 33.34%. The efficacy and reliability of the suggested encryption algorithm are confirmed by the average intensity values of NPCR and UACI. As summarized in Table 9, the proposed scheme achieves NPCR (99.5983%) and UACI (33.325%) (close to the 8-bit random-pair reference (33.46%)), indicating strong resistance to differential attacks.

### 5.7. Chosen Plain-Text Image Analysis

It is used to test the strength of an image encryption algorithm when an attacker tries to select the all-black, all-white, or any image with a clear pattern and observe the corresponding encrypted image. An attacker chooses a simple image and analyzes how it is transformed into the encrypted image, as research can verify whether the encryption algorithm effectively hides the pixel pattern and structural look. A secure and strong encryption algorithm always ensures that even the selection of these types of simple images cannot help the attacker extract any meaningful information or remove all clues for the attacker to guess the encryption key and recover the original image.

**Figure 13:**
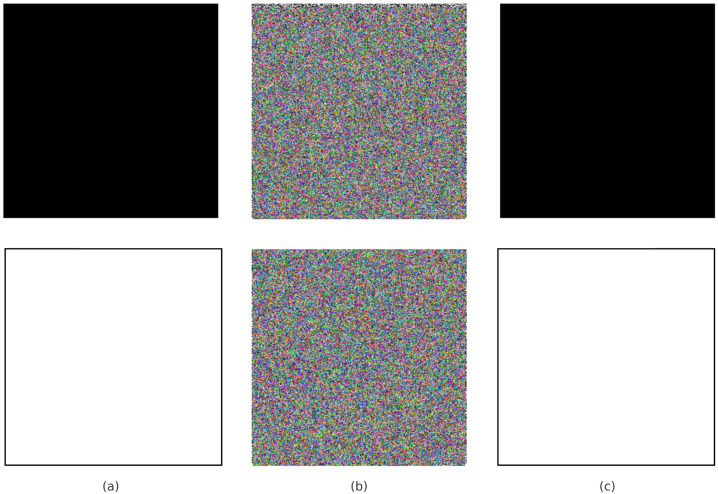
Encryption and decryption of all-black and all-white images: (**a**) Original plain images (all-black and all-white); (**b**) Corresponding cipher images after encryption; (**c**) Decrypted images recovered from the cipher images.

Figure 13 illustrates the chosen plain-text analysis, where we selected all-black and all-white images. In the encryption phase, the plain images are completely transformed into the cipher image, which is highly random with uniform noise, making it impossible to extract any meaningful information. This ensures that the proposed encryption algorithm is not dependent on the variety of input images and can effectively encrypt the image even with minimal pixel variation. As shown in Table 10, the proposed cipher maintains high NPCR and near-ideal UACI values for both all-black and all-white images, demonstrating robustness against chosen-plaintext attacks.

### 5.8. Computational Efficiency and Time Complexity

#### 5.8.1. Experimental Setup

All the measurements were obtained on an *Asus Vivobook* equipped with an AMD Ryzen 9 processor, 32 GB RAM, and an AMD Radeon graphics adapter. The LSTM was trained using 80% of the chaotic trajectories for 500 epochs. Table 11 reports the measured training, inference, encryption, and decryption times, confirming that the proposed scheme operates efficiently with near real-time performance on standard hardware.

#### 5.8.2. Algorithmic Time Complexity

Let $P\in Z_{8}^{M\times N\times 3}$ denote an M×N color image. Each encryption stage performs a constant number of linear passes: (i) pseudorandom matrix generation (length MN) (ii) first and second scramblings (Fisher–Yates/Thorp swap/rotate; sub-image blocking, zigzag, rotation, and permutation) are each O(MN); (iii) DNA encode/operate/decode (channelwise) is O(MN); (iv) inverse steps mirror the same costs. Thus, the cipher’s end-to-end complexity is O(MN) per image (and per decryption), exclusive of LSTM. For the LSTM, let *L* be the predicted window length and $d_{h}$ the hidden width; standard gated-cell inference has cost $O(L,d_{h}^{2})$ for dense implementations. Training is a one-time offline step; inference can be executed offline to pre-buffer keystreams.

#### 5.8.3. Discussion

The permutation-diffusion cipher is linear-time and completes within a few seconds for 256 × 256 images on commodity hardware. The LSTM stage is trained once and does not need to be repeated during deployment; its inference can be performed offline (e.g., batched keystream preparation) so that the online path is dominated by the O(MN) cipher passes. These characteristics indicate practical scalability for clinical image sizes; GPU-optimized inference or distilled recurrent architectures can further reduce the amortized latency of keystream preparation when required.

### 5.9. Experimental Environment and Reproducibility

#### 5.9.1. Hardware

All the experiments were executed on an Asus Vivobook laptop equipped with an AMD Ryzen 9 processor, 32 GB RAM, and an AMD Radeon graphics adapter.

#### 5.9.2. Software Stack

The implementation uses a standard scientific Python environment: Python 3.10 with NumPy 1.24.4, SciPy 1.10.1, and scikit-image 0.20.0 for array and image operations, and Matplotlib 3.7.1 for plotting. The LSTM was implemented in a mainstream deep learning framework (PyTorch 1.13.1, CPU backend). Numerical ODE integration for the 4D chaotic driver and Lyapunov exponent estimation followed SciPy-based routines. Random seeds were fixed for Python and NumPy to ensure run-to-run reproducibility.

#### 5.9.3. Conclusions

In this work, we implemented two scrambling operations and a DNA-based diffusion process to present a novel algorithm for medical image encryption and decryption. Initially, we utilized four four-dimensional chaotic systems and seeded their chaotic sequences into the Long Short-Term Memory (LSTM) model for training and predicting new chaotic sequences. After this, we generated the pseudorandom matrices, which helped us to add confusion in the image pixels. The combination of these techniques ensures a high level of randomness and complexity in the encrypted image, which provides it with strong resistance against brute-force attacks.

The security analysis, including high entropy, low correlation value, NPCR, and UACI, and histogram and robustness analysis, ensures that the proposed encryption algorithm eliminates any kind of relation or pattern between adjacent pixels in the encrypted image. The results of the security analysis further ensure that even under unintentional data loss or Gaussian noise interference, the encryption scheme maintains its structure, highlighting its robustness in real-world applications. Furthermore, a sufficiently large value of the key space, $2^{384}$, analysis reveals that the encryption algorithm has an extremely large key space, indicating resistance against differential and statistical attacks.

In the fields of medical and multimedia images, the proposed encryption algorithm provides a secure, efficient, and robust framework suitable for protecting sensitive image data. The proposed scheme holds promise for securing practical applications, with its strong resistance against statistical and differential attacks, including robustness against data loss. Future work can extend this approach to video encryption and real-time image transmission, ensuring secure communication in broader multimedia systems. 

## Figures and Tables

**Table 1:** DNA encoding rules.

Rule	00	01	10	11
1	A	C	G	T
2	A	G	C	T
3	C	A	T	G
4	C	T	A	G
5	G	A	T	C
6	G	T	A	C
7	T	C	G	A
8	T	G	C	A

**Table 2:** DNA operations under encoding rule 01 (00 ↦ A, 01 ↦ C, 10 ↦ G, 11 ↦ T).

+	A	T	C	G	−	A	T	C	G	⊕	A	T	C	G
A	A	T	C	G	A	A	C	T	G	A	A	T	C	G
T	T	G	A	C	T	T	A	G	C	T	T	A	G	C
C	C	A	G	T	C	C	G	A	T	C	C	G	A	T
G	G	C	T	A	G	G	T	C	A	G	G	C	T	A

**Table 3:** Key space analysis comparison.

Algorithm	Key Space
Proposed	$2^{384}$
Zhou et al. [29]	$2^{279}$
Su et al. [22]	$2^{227}$
Dai et al. [28]	$2^{388874304}$
Niu et al. [16]	$10^{60}$
Patel et al. [26]	$2^{128}$
Zhao et al. [37]	$2^{509}$

**Table 4:** Entropy analysis results for encrypted medical images.

Image	Size	Entropy R	Entropy G	Entropy B
Brain-tumor-01	256×256	7.9902	7.9987	7.9913
Brain-tumor-02	256×256	7.9962	7.9964	7.9973
Brain-tumor-03	256×256	7.9919	7.9990	7.9950
Brain-tumor-04	256×256	7.9971	7.9970	7.9965
Brain-tumor-05	256×256	7.9962	7.9964	7.9975
Brain-tumor-06	256×256	7.9970	7.9969	7.9976
Average	—	7.9944	7.9977	7.9959

**Table 5:** Entropy comparison.

Algorithm	Entropy
Proposed	7.9960
Khalil et al. [38]	7.9973
Wang et al. [13]	7.9970
Wei et al. [14]	7.9969
Liang et al. [39]	7.9972
Ding et al. [12]	7.9985
Raghuvanshi et al. [25]	7.9996
Kadir et al. [24]	7.9744

**Table 6:** Correlation coefficient analysis for original and encrypted images in horizontal (H), vertical (V), and diagonal (D) directions.

Channel	H-Orig	V-Orig	D-Orig	H-Enc	V-Enc	D-Enc
R	0.9915	0.9973	0.9890	−0.0001	−0.0033	0.0011
G	0.9915	0.9973	0.9890	0.0104	−0.0115	−0.0115
B	0.9915	0.9973	0.9890	−0.0142	−0.0019	−0.0152
R	0.9953	0.9737	0.9697	0.0022	0.0029	0.0079
G	0.9953	0.9737	0.9697	0.0086	0.0059	−0.0022
B	0.9953	0.9737	0.9697	0.0016	−0.0041	−0.0052
R	0.9966	0.9840	0.9811	0.0087	0.0109	0.0015
G	0.9966	0.9840	0.9811	0.0180	−0.0059	−0.0049
B	0.9966	0.9840	0.9811	0.0135	0.0091	0.0113
R	0.9881	0.9899	0.9792	0.0036	−0.0007	0.0139
G	0.9881	0.9899	0.9792	0.0033	0.0014	0.0046
B	0.9881	0.9899	0.9792	0.0053	−0.0043	0.0138
R	0.9432	0.9704	0.9162	0.0155	0.0036	0.0137
G	0.9432	0.9704	0.9162	0.0130	−0.0087	0.0044
B	0.9432	0.9704	0.9162	−0.0081	0.0035	−0.0026
R	0.9296	0.9601	0.9026	0.0119	0.0004	0.0045
G	0.9296	0.9601	0.9026	0.0016	0.0073	0.0096
B	0.9296	0.9601	0.9026	−0.0064	−0.0049	0.0034

**Table 7:** Correlation analysis of the proposed method compared with the existing schemes.

Direction	Proposed	[17]	[18]	[27]	[19]	[20]	[21]	[15]
Horizontal	−0.0001	−0.0023	−0.01317	0.0041	−0.0074	0.0194	−0.0093	0.0025
Vertical	−0.0033	−0.0042	0.00136	−0.0007	0.0019	0.0195	0.0025	0.0029
Diagonal	0.0011	−0.00354	0.00224	0.0002	−0.0017	0.0195	−0.0024	0.0027

**Table 8:** NPCR and UACI analysis for different test images across R, G, and B channels with average values.

Name & Size	Modified Pixel Position	NPCR_R	NPCR_G	NPCR_B	UACI_R	UACI_G	UACI_B
Brain-tumor-06	(42 11 57)	99.6368	99.6002	99.6490	33.6731	33.4912	33.6381
Brain-tumor-05	(122 77 212)	99.6124	99.6033	99.6216	33.5279	33.3474	33.4891
Brain-tumor-04	(181 17 118)	99.6170	99.6063	99.5895	33.5093	33.6441	33.5659
Brain-tumor-03	(101 100 100)	99.5667	99.5651	99.5865	34.3838	34.2267	33.5537
Brain-tumor-02	(21 34 111)	99.5834	99.6124	99.5926	33.4929	33.3945	33.3622
Brain-tumor-01	(177 22 219)	99.5117	99.5758	99.5987	34.4794	34.8643	33.8939
Average	—	99.5880	99.5939	99.6063	33.8444	33.8280	33.5838

**Table 9:** Comparison of NPCR and UACI values.

Algorithm	NPCR	UACI
Proposed	99.5983	33.325
Yang et al. [6]	97.3300	25.36
Huang et al. [5]	99.6100	33.4000
Zhuang et al. [7]	99.60	33.53
Malathy et al. [40]	42.4252	37.872
Kamal et al. [21]	99.6173	33.4755
Belazi et al. [15]	99.6010	33.4389

**Table 10:** Statistical results for all-black and all-white images.

	NPCR	UACI	Entropy	Correlation Coefficients		
				Horizontal	Vertical	Diagonal
All-black	99.6173	33.3474	7.9967	−0.0081	0.0035	0.0026
All-white	99.6010	34.2267	7.9945	−0.0013	−0.0023	0.0011

**Table 11:** Runtime measurements on Asus Vivobook (AMD Ryzen 9, 32 GB RAM, AMD Radeon).

Operation	Workload	Wall Time
LSTM training (one-time)	80% chaotic data, 500 epochs	≈90 min
LSTM inference (keystream)	65,536 values (256×256)	≈15 min
Encryption (cipher only)	256×256 color image	≈5.0 s
Decryption (cipher only)	256×256 color image	≈5.5 s

## Data Availability

The images used in this study were obtained from the Brain Tumor MRI Colorized Dataset available on Kaggle (https://www.kaggle.com/datasets/shuvokumarbasak2030/brain-tumor-mri-colorized-dataset), accessed on 15 June 2025. The dataset is released under the CC0: Public Domain license, permitting free use without restriction.
